# Fixation using alternative implants for the treatment of hip fractures (FAITH): design and rationale for a multi-centre randomized trial comparing sliding hip screws and cancellous screws on revision surgery rates and quality of life in the treatment of femoral neck fractures

**DOI:** 10.1186/1471-2474-15-219

**Published:** 2014-06-26

**Authors:** The FAITH Investigators

**Affiliations:** 1 Division of Orthopaedic Surgery, McMaster University, 293 Wellington Street North, Suite 110, L8L 8E7 Hamilton, ON, Canada

**Keywords:** Hip fractures, Femoral neck fractures, Fracture fixation, Internal fixation, Cancellous screws, Sliding hip screw

## Abstract

**Background:**

Hip fractures are a common type of fragility fracture that afflict 293,000 Americans (over 5,000 per week) and 35,000 Canadians (over 670 per week) annually. Despite the large population impact the optimal fixation technique for low energy femoral neck fractures remains controversial. The primary objective of the FAITH study is to assess the impact of cancellous screw fixation versus sliding hip screws on rates of revision surgery at 24 months in individuals with femoral neck fractures. The secondary objective is to determine the impact on health-related quality of life, functional outcomes, health state utilities, fracture healing, mortality and fracture-related adverse events.

**Methods/Design:**

FAITH is a multi-centre, multi-national randomized controlled trial utilizing minimization to determine patient allocation. Surgeons in North America, Europe, Australia, and Asia will recruit a total of at least 1,000 patients with low-energy femoral neck fractures. Using central randomization, patients will be allocated to receive surgical treatment with cancellous screws or a sliding hip screw. Patient outcomes will be assessed at one week (baseline), 10 weeks, 6, 12, 18, and 24 months post initial fixation. We will independently adjudicate revision surgery and complications within 24 months of the initial fixation. Outcome analysis will be performed using a Cox proportional hazards model and likelihood ratio test.

**Discussion:**

This study represents major international efforts to definitively resolve the treatment of low-energy femoral neck fractures. This trial will not only change current Orthopaedic practice, but will also set a benchmark for the conduct of future Orthopaedic trials.

**Trial registration:**

The FAITH trial is registered at ClinicalTrials.gov (Identifier NCT00761813).

## Background

Hip fractures are a common type of fragility fracture that afflict 293,000 Americans (over 5,000 per week) and 35,000 Canadians (over 670 per week) annually [1,2]. The prevalence of hip fractures is likely to exceed 500,000 annually in the United States and 88,000 in Canada over the next 40 years [3,4]. The estimated annual health care costs will reach a staggering $17 billion in the United States [5] and $650 million in Canada [6,7]. Hip fractures are associated with a 30% mortality rate at 1 year [8] and profound temporary and sometimes permanent impairment of independence and quality of life [9,10]. The disability-adjusted life-years lost as a result of hip fractures ranks in the top 10 of all cause disability globally [3].

Intracapsular fractures (also known as femoral neck fractures) may be either undisplaced or displaced. Internal fixation, in which a mechanical implant fixes the two segments of bone together, is the best way to manage undisplaced fractures [11] and, depending on patient characteristics and surgeon preference, can be used to manage displaced fractures as well [12].

Complications of internal fixation include avascular necrosis of the femoral head as well as early implant failure and nonunion [13,14]. Approximately 30% of surgically treated hip fractures require revision surgery [15-17]. These revisions are associated with a large burden of morbidity and appreciable mortality. Even patients who do not require revision surgery may have long term functional limitation due to soft tissue damage at the time of surgery.

The most popular approaches to internal fixation include multiple cancellous screws or a single compression screw and side plate (i.e., sliding hip screws). Both approaches have strong physiologic rationale and ardent advocates; however, there is no consensus regarding the optimal approach for management of intracapsular hip fractures [12].

### Multiple cancellous screws

Advocates of multiple cancellous screws focus upon its superior torsional stability, limited disruption of femoral head blood supply, and minimally invasive insertion [18-22]. Based on physiologic and observational studies, most surgeons use 3 parallel cancellous screws in a triangular (typically inverted) orientation [12,20]. Proponents of cancellous screws further argue that small diameter screws retain more viable bone after insertion compared to the larger sliding hip screws [18-22]. Retaining more cancellous bone optimizes vascularity and thus may reduce the risk of avascular necrosis of the femoral head. A randomized controlled trial (RCT) comparing 3 cancellous screws with a larger sliding hip screw found 3.5-fold greater femoral head vascularity at follow-up bone scanning with screws [22]. Surgeons can insert cancellous screws using 3 small stab incisions with limited blood loss and operating time. This minimally invasive approach may limit damage to the soft tissues around the hip and plausibly lead to better patient function and general wellbeing.

### Sliding hip screws

Despite the popularity of cancellous screw fixation, there is a strong biologic rationale supporting the use of sliding hip screws, a more biomechanically stable construct, in older patients with osteopenia or osteoporosis [23]. Cancellous screw fixation improves torsional strength; however, implant failures typically occur with bending and vertical shear loads (i.e., with weight-bearing). The sliding hip screw, the gold standard approach in treating inter-trochanteric fractures of the hip, has gained popularity as an alternative in the management of femoral neck fractures. Proponents of the sliding hip screw believe its superior biomechanical properties and greater fracture stability in osteoporotic bone should decrease the need for revision surgery. Sliding hip screw constructs have shown 2-fold greater maximal strength and less displacement under physiologic loading conditions compared to cannulated screws. In a cadaveric model, the sliding hip screw performed better than cancellous screws in stabilizing unstable femoral neck fractures under cyclic loading [21]. The sliding hip screw performed better in osteoporotic bone and was less sensitive to decline in bone mineral density than screws [21]. Newer minimally invasive techniques allow sliding hip screws to be inserted with small incisions and smaller side plates with limited blood loss and operating times [24].

### Inconclusive clinical evidence

Bhandari and colleagues conducted a systematic review and meta-analysis comparing arthroplasty to internal fixation in patients with displaced femoral neck fractures. Results demonstrate that while it may increase surgical mortality, arthroplasty reduced the need for revision surgery (RR = 0.23, 95%; CI: 0.13-0.42, homogeneity p < 0.01) [16]. The authors conducted a sensitivity analysis to explore reasons for variability and found that arthroplasty, compared to internal fixation, appeared to decrease the risk of revision far more when the method of internal fixation was screws alone (RR = 0.11) than when the method of internal fixation was a compression screw and side plate (RR = 0.59) (p < 0.01 for difference in estimates).

A meta-analysis by Parker and colleagues evaluated 28 trials (N = 5547 patients) and reported no advantage of any internal fixation technique over any other technique [25]. The trials were small (range in sample sizes: 33 to 410), and methodologically limited. Only 5 RCTs evaluated sliding hip screws versus cancellous screws (4 displaced femoral neck fractures, 1 undisplaced femoral neck fractures). These trials ranged in size from 33 to 209 patients and had few total outcome events (range: 3–34 events). When the data from these 5 studies were pooled, the point estimate trended toward favouring sliding hip screws compared to cancellous screws; however, the difference was not statistically significant.

In summary, both direct comparisons between sliding hip screws and cancellous screws and indirect comparisons (comparing sliding hip screws and cancellous screws separately to arthroplasty) suggest a possible benefit for a sliding hip screw over multiple cancellous screws in reducing the need for revision surgery. The indirect nature of the comparison from the meta-analysis of arthroplasty versus internal fixation, and the small sample sizes, methodological limitations, and non-significant pooled estimates from the direct comparisons, leaves the issue very much in doubt.

### Surgical management of hip fractures

Bhandari and colleagues contacted 442 surgeons in an international survey [12] to explore surgeons’ opinions regarding internal fixation for femoral neck fractures. For undisplaced femoral neck fractures, 92% of surgeons preferred internal fixation. Despite the evidence suggesting the superiority of sliding hip screws, 90% of surgeons preferred cancellous screws. In displaced hip fractures, 25% of surgeons preferred internal fixation when compared with arthroplasty; and of those who preferred internal fixation, 68% preferred cancellous screws.

## Methods/Design

### Pilot and vanguard phase

We have successfully completed a pilot RCT (n = 80 patients) to assess the feasibility of the definitive FAITH trial. The primary outcomes of the pilot study were the rates of reoperation at 24 months to promote fracture healing, relieve pain, treat infection, treat a peri-prosthetic fracture, or improve function. We also collected functional outcomes on all patients. Our pilot RCT demonstrated: (1) our ability to recruit patients for the definitive trial; (2) investigator compliance with key aspects of the protocol; (3) maintenance of data quality; (4) maintenance of high follow-up rates; (5) our ability to organize trial procedures in a multinational trial; and (6) overall event rates for our primary outcome that support our sample size calculation. We have also used the vanguard phase to optimize case report forms and a manual of operations. To avoid a break in funding and to maintain our momentum across recruiting sites, the Vanguard phase transitioned into the definitive trial phase without stopping. The rationale for the definitive FAITH trial is supported by: (1) the substantial burden of hip fracture injuries to both patients and the healthcare system; (2) differences in the surgical management of hip fractures; (3) a lack of clinical evidence to inform the optimal method of internal fixation; (4) the demonstrated success of the pilot RCT.

### Study objectives

The primary objective of the FAITH study is to evaluate the effects of sliding hip screws versus cancellous screws on revision surgery at 24 months post-injury. The secondary objectives are to determine the impact of sliding hip screws versus cancellous screws on health-related quality of life, functional outcomes, health state utilities, fracture healing, mortality, and other adverse events such as avascular necrosis, nonunion, malunion, implant breakage or failure, and infection.

### Study design

FAITH is a multi-centre, concealed RCT using minimization to determine patient allocation. We will randomly assign a minimum of 1,000 patients who have sustained a femoral neck fracture to one of two treatment groups. The first treatment group involves fixation of the fracture with multiple small diameter cancellous screws (i.e., cancellous screw group). The second treatment group involves fixation of the fracture with a single larger diameter screw with a side plate (i.e., sliding hip screw group). We hypothesize that sliding hip screws will have lower rates of re-operation (primary outcome) and higher functional outcome scores (secondary outcome) at 24 months when compared with cancellous screws. The FAITH trial is registered at ClinicalTrials.gov (Identifier NCT00761813) and has received McMaster University Research Ethics Board approval (REB#: 06–402) and approval from local ethics boards.

### Randomization

We will randomize patients using minimization to ensure balance on baseline covariates An automated Internet-based randomization system centralized at the FAITH Methods Centre at McMaster University, which we have used successfully for other multicenter trials, will ensure concealed randomization of eligible consenting patients. Concealed randomization will be ensured through two strategies: 1) the randomization sequence will be predetermined by research personal who are external to the clinical sites who are determining patient eligibility; and 2) the internet-based randomization system will require surgeons to enter patients into the trial before treatment allocation is divulged. Minimization will be used to ensure balance between intervention groups for several patient factors. The minimization approach takes into account each pre-identified prognostic variable and sums over the variables to allocate each patient to the treatment that minimizes the current differences between groups for those prognostic variables. Unlike stratified randomization, minimization works toward minimizing the total imbalance for all factors together instead of considering mutually exclusive subgroups (strata) [26]. Based upon our international survey of surgeons [12] and current evidence [27], we will minimize for the following prognostic factors: (1) Age (i.e., 50–80 years or >80 years (81 years or older)); (2) Undisplaced or displaced femoral neck fractures; (3) Pre-fracture living setting (i.e., institutionalized or not institutionalized); (4) Pre-fracture functional status (i.e., using ambulatory aid or independent ambulator); (5) American Society for Anesthesiologists (ASA) Class (i.e., Class I/II or III/IV/V) [28]; and (6) Centre.

### Eligibility criteria

Eligible patients will meet all the following inclusion criteria: (1) Men or women aged 50 years and older (with no upper age limit); (2) Fracture of the femoral neck confirmed with anteroposterior and lateral hip radiographs, computed tomography, or magnetic resonance imaging (MRI); (3) Operative treatment of displaced fractures within 4 days of presenting to the emergency room, or operative treatment of undisplaced fractures within 7 days of presenting to the emergency room; (4) Patient was ambulatory prior to fracture, though they may have used an aid such as a cane or a walker; (5) Anticipated medical optimization for operative fixation of the hip; (6) Provision of informed consent by patient or a legally appointed representative; (7) No other major trauma (defined as an Injury Severity Score >16); and (8) Low energy fracture, in the judgment of the attending surgeon.

We will exclude patients meeting any of the following criteria: (1) Patients not suitable for internal fixation (i.e., severe osteoarthritis, rheumatoid arthritis, or pathologic fracture); (2) Associated major injuries of the lower extremity (i.e., ipsilateral or contralateral fractures of the foot, ankle, tibia, fibula, knee, or femur; dislocations of the ankle, knee, or hip; or femoral head defects or fracture); (3) Retained hardware around the affected hip; (4) Infection around the hip (i.e., soft tissue or bone); (5) Patients with disorders of known bone metabolism except osteoporosis (i.e., Paget’s disease, renal osteodystrophy, osteomalacia); (6) Patients with a history of frank dementia that would interfere with assessment of the primary outcome (i.e., revision surgery at 24 months); (7) Likely problems, in the judgment of the investigators, with maintaining follow-up (i.e., patients with no fixed address, report a plan to move out of town, or intellectually challenged patients without adequate family support); and (8) Exclusion of a patient because of enrolment in another ongoing drug or surgical intervention trial will be left to the discretion of the attending surgeon, on a case-by-case basis.

### Patient recruitment and screening

**Figure 1:**
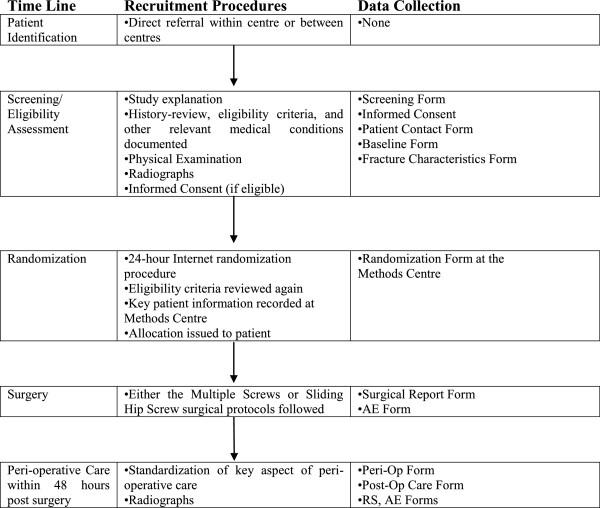
Recruitment schedule (Baseline radiographs & Data collection).

Data will be collected from academic hospitals in Australia, Canada, England, Germany, India, Norway, The Netherlands, and The United States. A complete list of clinical sites can be obtained from the corresponding author upon request. All patients presenting to participating surgeons with a diagnosed femoral neck fracture will be screened. Figure 1 shows the patient identification and screening procedures. Informed consent will be obtained from all eligible patients by qualified clinical site personnel who are knowledgeable about the study. If a patient lacks capacity and is deemed unable to consent, informed consent may be obtained from the patient’s legally authorized representative.

We will enroll all patients who meet the eligibility criteria and document reasons for failure to randomize all eligible patients. We will also document all patients screened for eligibility and record them as: (1) Eligible and included; (2) Eligible and missed; or (3) Excluded. The Central Adjudication Committee (CAC) will adjudicate all situations where eligibility is in doubt.

### Study interventions

#### *Cancellous screw*

Patients allocated to the cancellous screw group will receive multiple threaded screws (with a minimum of 2 screws and a minimum diameter of 6.5 mm). Surgeons will be allowed to use any threaded screw or hook pin (i.e., Gouffon, Uppsala, von Bahr, Hansson hook pins, etc.) or cancellous threaded screw (i.e., cannulated or non-cannulated, Ace, AO/Anklylosing Spondylitis International Federation (ASIF), Asnis, Richards, etc.). Surgeons will follow the technique guides associated with the screw manufacturers. No injectable bone substitutes will be allowed for augmentation of the implant fixation. Surgeons will document the following: (1) Number of screws; (2) Number of washers; (3) Manufacturer; (4) Reduction technique; (5) Decision to perform a capsulotomy or aspirate intracapsular hematoma; and (6) Screw configurations, especially of the third screw (outside of 2 critical placements inferiorly and posteriorly).

#### *Sliding hip screw*

Patients allocated to the sliding hip screws group will receive a single larger diameter partially threaded screw affixed to the proximal femur with a side plate (with a minimum of 2 holes and a maximum of 4 holes) and no supplemental fixations. Surgeons will be permitted to use any commercially available sliding hip screw implant (i.e., Stryker, DePuy, Synthes, Smith and Nephew, Zimmer, etc.), and will insert implants as per the manufacturers’ technical guides. The derotational kirschner wire should penetrate the acetabulum to provide maximal resistance to torsion. A centre-to-centre approach is recommended, while avoiding a superior and anterior approach. Spiral blades and helical screws are permitted, because they function similarly to the sliding hip screw. It will be documented when spiral blades and supplemental/derotational screws are employed by the surgeon.

The use of a compression screw, manufacturer, reduction technique, decision to perform a capsulotomy, aspiration of intracapsular hematoma, and final screw position measured by the Tip Apex Distance will be documented and based upon surgeon preference. No injectable bone substitutes will be allowed for augmentation of the implant fixation.

### Standardization of procedures and peri-operative care

We will recommend that clinical sites standardize key aspects of peri-operative care including: (1) Pre-operative antibiotics (i.e., cephalosporins, or equivalent coverage) administered before surgery and continued for 24 hours post-operatively; (2) Pre-operative thromboprophylaxis (i.e., Thromboembolic Disease (TED) Stockings, pneumatic compression boots, or medical prophylaxis discontinued in sufficient time to allow surgery as guided by International Normalized Ratio (INR)/Partial Thromboplastin Time (PTT)); (3) Post-operative thromboprophylaxis with unfractionated Heparin, Low Molecular Weight Heparin (LMWH), Warfarin, anti-platelet agents, or intermittent pneumatic compression boots.

Post-surgery, we will recommend that patients will be weight-bearing as tolerated and then advanced according to the attending surgeon’s best judgment (i.e., we will permit touch weight-bearing for displaced fractures and then advanced weight-bearing according to the surgeon’s best judgment).

We will recommend that all patients in the study receive 600 mg of Calcium by mouth (PO) daily and/or vitamin D 1000 International Units (IU) per day (provided there are no contraindications), or further investigation and treatment of osteoporosis as recommended by a local osteoporosis expert/consultant as necessary. Appropriate nutritional assessment with administration of oral micronutrient feeds will also be provided as needed.

Patient positioning, fracture reduction, and surgical exposure in the operating room will not be standardized as these are highly variable across the world. However, to ensure similar peri-operative care regimens it will be recommended that participating centres standardize these aspects of patient care. Finally, due to a lack of evidence favouring a particular approach, the following will be recorded but not standardized: (1) Use of pre-operative traction; (2) Surgical delay; (3) Type of anesthetic (i.e., general versus regional); and (4) Physiotherapy and rehabilitation programs. All patients in the study will receive medical consultation to optimize condition prior to surgery.

### Study outcomes

#### *Primary study endpoints*

The primary study endpoint is re-operation within 24 months post initial surgery to promote fracture healing, relieve pain, treat infection, or improve function including the following: (1) Implant removal prior to fracture healing; (2) Revision surgery with another internal fixation implant; (3) Revision surgery to arthroplasty; and (4) Soft tissue procedures, especially incision and drainage for deep infection at the bone implant interface. Classification of the reason for revision surgery is as follows: (1) Implant failure (2) Superficial or deep infection ; (3) Avascular necrosis; (4) Hip instability; (5) Hip dislocation; (6) Open wound; (7) Painful hardware; (8) Intractable pain due to wear of the acetabulum; (9) Peri-prosthetic femur fracture; and (10) Nonunion. Other classifications will be used if applicable. Planned revision surgeries will not be considered study events (i.e., technical malreduction requiring early revision). Criteria for the diagnosis of nonunion will include a failure of the fracture to progress towards healing for at least 2 months on consecutive radiographs. This will be evident as a persistent fracture line on radiograph, continued pain with hip range of motion, and the inability to weight-bear without pain. Avascular necrosis will be defined as a process that is characterized pathologically by bone marrow ischemia and eventual death of trabecular bone. Infection will be classified according to the Center for Disease Control Criteria [29].

Our choice of a 24 month follow-up period is dictated by the following 2 factors: (1) We have established a consensus among participating surgeons that the decision to re-operate on the hip fracture would occur within the first 24 months following surgery in all, or virtually all, patients and (2) Previous studies have reported 100% of revision surgeries occurring between 2 and 12 months for undisplaced fractures and 70-100% in 24 months for displaced fractures. Although nonunion and avascular necrosis may be present after 24 months, previous studies suggest that 70-98% of revisions due to nonunion and avascular necrosis occur within 24 months [30,31]. Thus, we can expect that nearly all the revision surgeries will occur within 24 months.

#### *Secondary study endpoints*

The secondary study endpoints include: (1) Health-related quality of life; (2) Functional outcomes; (3) Health state utilities; (4) Fracture healing; (5) Mortality; and (6) Other adverse events such as avascular necrosis, non-union, malunion, implant breakage or failure, and infection. Health-related quality of life will be measured by the Short Form-12 (SF-12) [32], the EuroQol-5 Dimensions (EQ-5D) [33], and the Western Ontario and McMaster Universities Arthritis Index (WOMAC) [34].

The SF-12 is a 12-item questionnaire that measures health-related quality of life across 8 domains. Both physical and mental summary scores can be obtained. Each domain is scored separately from 0 (lowest level) to 100 (highest level). The instrument has been extensively validated and has demonstrated good construct validity, high internal consistency, and high test-retest reliability [35] and is frequently used in orthopedics for evaluating fracture outcomes.

The WOMAC index is self-administered and assesses the three dimensions of pain, disability and joint stiffness in knee and hip osteoarthritis using a battery of 24 questions [34]. It is a valid, reliable and responsive measure of outcome, and has been used in several studies involving a wide range of lower extremity conditions [36].

The EQ-5D is a comprehensive, compact health status classification and health state preference system [33]. This questionnaire is widely used and has demonstrated validity and sensitivity in many populations [33,35].

### Adjudication of study events

The CAC is comprised of a Chairperson plus three orthopaedic surgeon committee members. The CAC is responsible for assessing patient eligibility (if in doubt) and they will independently adjudicate radiographic characteristics and quality of the surgery, fracture healing, revision surgeries, fracture-related complications (i.e., avascular necrosis, nonunion, malunion, implant breakage or failure, infection), and mortality. The adjudicators may review the patients’ case report forms and x-rays and determine if the reported outcome meets the criteria for being a study event. Any disagreements between the adjudicators will be discussed and resolved during consensus conference calls with the CAC. If consensus cannot be reached after extensive discussion, a vote will be permitted at the discretion of the Chair. All decisions made by the committee will be final. The CAC members will not be blinded to treatment as it is not possible to accurately judge blinded images; however, they will be blinded to the name and location of the clinical site.

### Study follow-up

**Figure 2:**
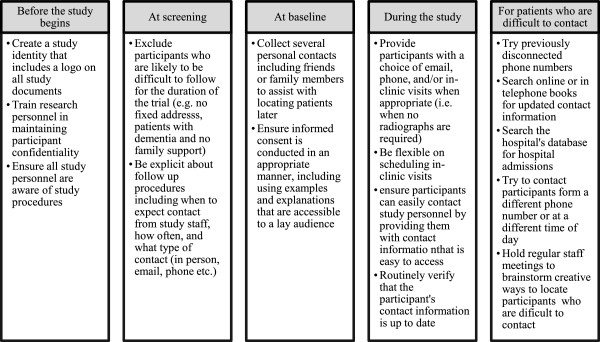
**Limiting loss to follow-up; Reference: Sprague S, Leece P, Bhandari M, Tornetta P, Schemitsch E, Swiontkowski M, SPRINT Investigators.** Limiting loss to follow up in a multicentre randomized controlled trial in orthopaedic surgery. Controlled Clinical Trials. 2003;24:719–725.

Table 1 shows the schedule of study follow-up events. At each follow-up, study outcomes will be recorded. Any revision surgeries and adverse events will be recorded at each visit. Missed follow-up visits or early withdrawal will also be documented.We will only withdraw patients for the following scenarios: (1) Patients withdraw consent for participation; or (2) Patients are deemed lost to follow-up after the 24 month visit is overdue and all exhaustive measures have been taken to locate the patient (see Figure 2 for a detailed depiction of methodology for limiting loss to follow-up). We will document the reasons for patient withdrawal from the trial. For those patients who withdraw from other study activities, we will seek their approval to collect clinical data from their medical and hospital charts, and/or to contact them by telephone to ask about the primary and secondary outcomes. We will not withdraw patients even if the study protocol was not adhered to (e.g., patient received wrong treatment arm, occurrence of protocol deviations, missed follow-up visits, etc.).

**Table 1:** Schedule of events

**Radiographs & event forms**	**Pre-surgery**			**Surgery**	**Post-surgery**								
	**Screening**	**Enrolment**	**Baseline**	**(Day 0)**	**≤ 48 hrs Post surgery**	**D/C**	**1 wk (24 hrs-10 ds)**	**10 wk (8-12 wks)**	**6 mo (5-7 mos)**	**9 mo (8-10 mos)**	**12 mo (11-13 mos)**	**18 mo (17-19 mos)**	**24 mo (≥24 mos)**
**Radiographs**			**●**		**●**			**●**			**●**		**●**
**Screening**	**●**												
**Informed consent**		**●**											
**Randomization**		**●**											
**Baseline**			**●**										
**Patient contact**		**●**			**●**	**●**	**●**	**●**	**●**	**●**	**●**	**●**	**●**
**Pre-Op care**			**●**										
**Fracture characteristics**			**●**										
**Surgical report**				**●**									
**Post-Op care**					**●**								
**Clinic or telephone follow-up**							**●**	**●**	**●**	**●**	**●**	**●**	**●**
**RS**					*		*	*	*	*	*	*	*
**AE**				*	*		*	*	*	*	*	*	*
**Hospital D/C**						**●**	*	*					
**Missed**													
**Follow-up**						*	*	*	*	*	*	*	
**Early W/D**						*	*	*	*	*	*	*	*

*Complete forms when and/or if applicable.

### Protecting against sources of bias

#### *Blinding*

In the FAITH trial surgeons cannot be blinded since the implants for the two treatment arms are easily distinguishable. Patients cannot be blinded because resulting surgical scarring patterns from both fixation procedures indicate which treatment was performed. Additionally, some patients and/or their caregivers often wish to review x-rays during clinic follow-up visits. Outcome assessors cannot be blinded as blinded images create difficulty in accurately adjudicating patient eligibility, radiographic characteristics, surgical quality, healing, and study events. Data analysts will be blinded.

#### *Surgeon expertise*

Similarly to other trials that evaluate two or more surgical interventions, there is a risk of differential expertise bias [37]. However, training in both methods of internal fixation examined in FAITH are standard training for Orthopaedic surgeons. We will also recommend that surgeons participating in the FAITH trial should meet 2 criteria for expertise for either cancellous screw fixation or sliding hip screw fixation: (1) Surgeons should have performed at least 25 procedures (of either sliding hip screws and/or cancellous screws) in their career (including residency experience in which they assumed responsibility for the procedure); and (2) Surgeons should continue to perform the procedure (at least 5 per year of either sliding hip screws and/or cancellous screws) in the year prior to the trial start date. Additionally, we will standardize aspects of the peri-operative care in order to limit any potential expertise bias and differential use of interventions. With these two procedures in place it is unlikely that a significant degree of expertise bias will be introduced into the trial.

### Maximizing patient retention

Previous trials in hip fracture surgery have lost up to 50% of patients to follow-up [16]. To avoid this problem we will implement several procedures, as outlined in (Figure 2). We have previously used the majority of these strategies to maximize follow-up in multi-centre studies. Key tactics include: (1) Excluding individuals who are likely to present problems with follow-up; (2) Prior to hospital discharge, as well as their own telephone number, each patient will provide the name and address of alternate contacts who are likely to be aware of the patient’s whereabouts; (3) Patients will receive a reminder card for their next follow-up visit from the clinical research coordinator; and (4) Follow-up visits will coincide with standard fracture clinic visits. Alternatively, patients can complete some study visits over the phone for the visits where no radiographs are required. Additionally, we will not withdraw patients if the study protocol was not adhered to (e.g. patient received wrong treatment arm, occurrence of protocol deviations, missed follow-up visits, etc.).

### Minimizing co-intervention and contamination

Any patients who crossover will be analyzed in the group to which they were allocated, maintaining the intention to treat approach for the analysis. It remains possible that surgeons may initially plan upon internal fixation but decide intra-operatively to replace the hip with some form of arthroplasty (i.e., hemi-arthroplasty or total arthroplasty). These patients, who never receive either internal fixation implant, will be excluded from the analysis if the decision to choose arthroplasty could not have plausibly been related to the group to which the patient was randomized. This decision will be made by the CAC. In previous trials of internal fixation versus arthroplasty, 14% of patients randomized to internal fixation implants actually received an arthroplasty [38]. The intraoperative switch to arthroplasty procedures when internal fixation is planned is related to surgeon biases for a particular procedure and the inability to obtain a satisfactory reduction of the femoral fracture for the placement of internal fixation.

Our standardization of management protocols will limit co-interventions, and we will document the use of drugs that affect the bone, and major additional procedures that patients undergo. Similarly, the use of medications that affect bone metabolism will be discouraged, but not prohibited. Research coordinators will record all medications such as a bisphosphonates, hormone replacement therapy, selective estrogen receptor modulators, calcitonin, and anabolic steroid therapy used concurrently.

### Statistical plan

#### *Sample size determination*

The determination of sample size is based upon a comparison for the primary outcome (revision surgery) of the sliding hip screw versus multiple cancellous screw groups. All statistical hypotheses will be assessed using 2-sided significance tests. Alpha levels of 0.05 have been chosen for the primary and 0.01 for the secondary outcomes. Previous studies have reported revision surgery rates in hip fracture patients that have ranged from 0-44% [39-41], with a weighted pooled risk of 22.7% (95% CI: 19.6-25.8%). In a pooled estimate of 5 RCTs comparing sliding hip screw fixation with multiple screw fixation in patients with displaced or undisplaced hip fractures, sliding hip screws reduced the risk of revision by 32% (95% CI: 3-53%, p = 0.04, Q = 5.3) [22,42-45]. The sample size calculation reflects the proposed primary analysis, which will use the Cox proportional hazards model, and is based on methods described by Collett [46]. The goal is to calculate the required number of patients that will yield a sufficient number of outcome events (revision surgeries) in order to have adequate statistical power for a given size of treatment effect. This was done by taking into account the anticipated revision surgery rates in the multiple cancellous screw group, the postulated relative risk reduction with sliding hip screws, and the rates of mortality and loss to follow-up. Because some of these inputs are expected to change over the two year period of follow-up, the expected number of person-years of follow-up and the expected numbers of study events in each group were calculated initially for the first year of follow-up (year 1). The calculations were then repeated for the second year of follow-up (year 2), after having estimated the number of patients in each group who would survive, be event-free, and available for continued follow-up at 12 months post-randomization.

The relative risk reduction was estimated to be 25% [43]. The decision to use a 25% annual event rate in the cancellous screws group for year 1 was based upon the previous literature [25,47] and compelling preliminary aggregate data (blinded to allocation) from our pilot study. Informed by the pilot study, annual mortality rates of 15% and losses to follow-up of 5% were assumed for each group. The annual event rates by group were converted into equivalent hazard rates, assuming for simplicity that the hazard rate would be approximately constant over year 1. It was calculated that approximately 58% and 63% of the group receiving cancellous screws and the group receiving sliding hip screw fixation would be event-free and available for further follow-up at the start of year 2. It was assumed that the revision rate would fall to one-third of its initial value during year 2 of follow-up (i.e., about 10% in the cancellous screws group), that the annual mortality rate would be 10%, and that there would again be 5% of patients lost to follow-up. The sample size was also increased to allow for an estimated combined 6.8% crossover rate from the assigned to the alternate treatment, based on our pilot data.

These assumptions lead to a required total sample size of 1,441 patients, who will yield an expected number of 353 outcome revisions. For convenience, the target number of patients was rounded to 1,500. Table 2 shows the study power for a sample size of 1,500 patients (750 per group) and a range of possible values for the initial revision rate and the relative risk reduction, on either side of the central assumed values. It demonstrates that with the chosen sample size of 750 per group, there will be a high likelihood of detecting a RRR of 25% or greater across the plausible range of revision surgery rates, and moderate power for slightly lower effect sizes.

**Table 2:** Estimated study power for 750 patients per treatment arm (N = 1500)

**Baseline risk**					
**(Year 1)^**	**Relative risk reduction**				
	**10%**	**15%**	**20%**	**25%**	**30%**
**20%**	15.9%	30.8%	50.5%	70.5%	**86.1%**
**25%**	19.6%	38.7%	61.6%	**81.5%**	**93.5%**
**30%**	23.6%	46.8%	71.7%	**89.3%**	**97.4%**
**35%**	28.2%	55.2%	**80.3%**	**94.5%**	**99.1%**
**40%**	33.3%	63.6%	**87.3%**	**97.5%**	**99.7%**

*Number of patients per treatment arm, alpha = 0.05.

^Year 2 risk is 1/3 that of year 1.

Bold numbers indicate statistical power range for this study.

After 589 patients completed study follow-up, event rates were obtained and used to inform power analyses for a reduced sample size of 1,000 patients. A sample size of 1,000 patients was used in order to obtain a balance between feasibility of completing recruitment within a reasonable timeline and obtaining a sample size that will support plausible hypotheses of treatment effect and baseline event rates. Based on event rates calculated from the first 589 patients who completed the study, a 15.5% annual event rate was used for year 1 and a 27.2% annual event rate was used for year 2. Based on the data from the completed patients, it was assumed that the annual mortality rate would be 11.0% (year 1) and 18.2% (year 2) and that there would be a 12.2% (year 1) and 5.9% (year 2) loss to follow-up. The sample size was again increased to allow for an estimated combined 6.8% crossover rate from the assigned to the alternative treatment. Table 3 shows the study power for a sample size of 1,000 patients (500 per treatment group) and a range of possible values for the initial revision rate and the relative risk reduction, on either side of the central assumed values. It demonstrates that with a sample size of 500 per group, there will be a likelihood of detecting a RRR of 30% or greater across the plausible range of revision surgery rates, and moderate power for slightly lower effect sizes.

**Table 3:** Estimated study power for 500 patients per treatment arm (N = 1,000)

**Baseline risk**							
**(Year 1)^**	**Relative risk reduction**						
	**10%**	**15%**	**20%**	**25%**	**30%**	**35v**	**40%**
**15.5%**	14.5%	28.5%	48.1%	69.3%	**86.3%**	**95.7%**	**99.2%**

*Number of patients per treatment arm, alpha = 0.05.

^Year 2 risk is 27.2%.

Bold numbers indicate statistical power range for this study.

While the revised assumptions based upon data collected from the first 589 patients to complete follow-up differ from the initial assumptions, the event annual rate of revision surgery of 15.5% in year 1 and 27.2% in year 2 are well within the range of 0-44% that have been previously reported in the literatures [39-41].

### Statistical methods

#### *Primary analysis*

All outcome analyses will adhere to the intention to treat principle. To evaluate the effect of sliding hip screw versus cancellous screws in time to revision rates, a Cox proportional hazards model will be used with the following covariates that are included in the minimization procedure: (1) Age (50–80 years or >80 years (81 years or older)); (2) Undisplaced or displaced femoral neck fractures; (3) Pre-fracture living setting (i.e., institutionalized or not institutionalized); (4) Pre-fracture functional status (i.e., using ambulatory aid or independent ambulator); (5) American Society for Anesthesiologists (ASA) Class (i.e., Class I/II or III/IV/V); and (6) Clinical site.

The treatment effect will be evaluated using a likelihood ratio test, and the validity of the proportional hazards assumption of the Cox model will be assessed. Survival rates at each follow-up period will be compared and then the overall difference between survival curves with log-rank tests will be evaluated, adjusting for the covariates. Absolute revision rates, risk reduction, absolute risk reduction, and the number needed to treat with screws and sideplate will be calculated. All results will be presented with 95% confidence intervals.

#### *Secondary analysis*

We will also examine the relative effect of sliding hip screw versus cancellous screws on time to mortality, avascular necrosis, nonunion, implant breakage or failure, time to fracture healing, and infection (i.e., superficial and deep) using Cox proportional hazards modeling. The treatment effect will be evaluated using a likelihood ratio test, and the validity of the proportional hazards assumption of the Cox model will be assessed. Survival rates at each follow-up period will be compared and the overall difference between survival curves with Log-Rank tests will be evaluated. Absolute revision rates, risk reduction, absolute risk reduction, and the number needed to treat with screws and side plate will be calculated. All results will be presented with 95% confidence intervals.

#### *Subgroup analyses*

We plan to conduct a single subgroup analysis comparing the effects of multiple screws versus sliding hip screw in patients with undisplaced and displaced femoral neck fractures. All analyses will be adjusted for important determinants of outcome (the prognostic variables outlined in the randomization section). The analysis plan is to fit logistic regression models and include treatment by subgroup interactions to assess whether the magnitude of the treatment effect is significantly different between subgroups. The survey and population-based data from Sweden suggests that two-thirds of all included fractures will be displaced [48]. Thus, approximately 1,000 displaced fractures (500 per group) and 500 undisplaced fractures (250 per group) can be expected. The weighted risk of revision in displaced fractures is 30.2%, (95% CI = 24.1, 6.4) and 20.4% (95% CI =17.4, 23.4%) in undisplaced fractures. The power tables suggest that the study will be able to detect relative risk reductions of 30% or greater with sliding hip screw compared to multiple cancellous screws in displaced fractures (Table 4).

**Table 4:** Displaced fractures: estimated study power for 500 patients per treatment arm (N = 1,000)

**Baseline risk**						
**(Year 1)^**	**Relative risk reduction**					
	**10%**	**15%**	**20%**	**25%**	**30%**	**40%**x
**20%**	12.0%	22.1%	36.3%	53.2%	70.1%	**93.0%**
**25%**	14.5%	27.6%	45.3%	64.5%	**81.0%**	**97.5%**
**30%**	17.2%	33.5%	45.3%	74.4%	**88.9%**	**99.2%**
**35%**	20.3%	40.0%	63.2%	**82.7%**	**94.1%**	**99.8%**
**40%**	23.8%	47.0%	71.7%	**89.2%**	**97.2%**	**100.0%**

*Number of patients per treatment arm, alpha = 0.05.

^Year 2 risk is 1/3 that of year 1.

Bold numbers indicate statistical power range for this study.

Previous randomized trials in patients with displaced fractures suggest that relative risk reductions with sliding hip screw as high as 48% are plausible [22,43-45]. Similarly, in undisplaced fractures the study will have at least 80% power to detect relative risk reductions of 40% or greater with sliding hip screws (Table 5).

**Table 5:** Undisplaced fractures: estimated study power for 250 patients per treatment arm (N = 500)

**Baseline risk**						
**(Year 1)^**	**Relative risk reduction**					
	**10%**	**15%**	**20%**	**25%**	**30%**	**40%**
**20%**	8.0%	13.2%	20.6%	30.3%	42.0%	68.0%
**25%**	9.3%	16.0%	25.5%	37.8%	51.3%	79.0%
**30%**	10.7%	19.1%	31.0%	45.6%	61.4%	**87.1%**
**35%**	12.3%	22.6%	36.3%	53.7%	70.3%	**92.7%**
**40%**	14.1%	26.5%	43.3%	61.8%	78.2%	**96.3%**

*Number of patients per treatment arm, alpha = 0.05.

^Year 2 risk is 1/3 that of year 1.

Bold numbers indicate statistical power range for this study.

Previous trials suggest risk reductions greater than 50% in undisplaced fractures [42,49]. If these previous estimates are correct, the current study will have adequate power for these analyses.

#### *Interim analysis*

An interim analysis will not be conducted. The trial will not be stopped early for benefit. The Data Safety Monitoring Board (DSMB) will monitor Adverse Events (AEs) and may make recommendations to the Principal Investigators and Steering Committee to stop the study for harm only.

### Data management

The Case Report Forms (CRFs) will be the primary data collection tool for the study. All data requested on the CRF must be recorded. An Electronic Data Capture system will be used to submit data to the Methods Centre located at McMaster University. Upon receipt of the data, the personnel at the Methods Centre will make a visual check of the data and they will query all missing data, implausible data, and inconsistencies.

### Ethical considerations

All patients will be provided a consent form describing the study and providing sufficient information to make an informed decision about their participation in this study. The consent form will be submitted with the protocol for review and approval by the REB/Institutional Review Board (IRB) for the study. The formal consent of a patient, using the REB/IRB-approved consent form, must be obtained before that patient undergoes any study procedure. Any amendments to the study protocol which may affect the conduct of the study, or the potential safety of or benefits to patients will require a formal amendment to the protocol requiring approval by McMaster University’s REB and local research ethics boards for clinical sites.

Information about study patients will be kept confidential and will be managed in accordance with the following rules: (1) All study-related information will be stored securely at the clinical site; (2) All study patient information will be stored in locked file cabinets and accessible only to study personnel; (3) All CRFs will be identified only by a coded patient number and initials; (4) All records that contain patient names, or other identifying information, will be stored separately from the study records that are identified only by the coded patient number and initials; and (5) All local databases will be password protected.

### Study committees

#### *Steering committee*

The Steering Committee is comprised of orthopaedic surgeons, a statistician, and research methodologists. The Steering Committee will provide guidance and direction to the overall trial.

### Data safety and monitoring board

The DSMB is comprised of Medical Doctors, a Safety Officer, and a Statistician who remain completely independent of the study investigators. The DSMB will review accumulated safety data from the trial and advise the Principal Investigators and the Steering Committee on items related to patient safety.

### Dissemination

Results from the primary manuscript will be submitted for publication regardless of whether or not there are significant findings. Every attempt will be made to ensure that the amount of time between completion of data collection and release of study findings are minimized.

Only the Methods Centre will have access to the full trial dataset. Data for the primary publication will be analyzed exclusively by the Methods Centre. Requests for access to the full trial dataset for secondary publications are encouraged and can be initiated through a written request to Methods Centre personnel.

## Discussion

Most of the current literature evaluating the efficacy of internal fixation at treating hip fractures is focused on the comparison between internal fixation and arthroplasty, which does not allow for a direct comparison of different methods of internal fixation. The RCTs that directly evaluate different types of internal fixation have been too small to definitively guide Orthopaedic practices as they lack sufficient power to reliably detect important treatment effects and some contain important methodological limitations [16,25]. FAITH will be the largest trial to date informing the question of the impact of cancellous screws and sliding hip screws on outcomes in patients with femoral neck fractures. With over 1000 patients, FAITH will have adequate power to detect important treatment effects and will help to inform Orthopaedic practice. FAITH will include patients from North America, Europe, Asia, and Australia making results generalizable to several different geographic populations.

Our previous trial evaluating reamed versus non-reamed nailing in over 1,300 patients with tibial shaft fractures (SPRINT) set a new benchmark for the conduct of RCTs in Orthopaedic surgery [50,51]. The FAITH trial expands upon the successful methodology employed in SPRINT and will set the bar higher as we expand our collaboration worldwide and recruit a larger number of patients, while maintaining methodological rigor. Ultimately, this RCT will move Orthopaedic surgical trials one step closer to the standards of global impact RCTs that are currently conducted in cardiovascular medicine and cerebrovascular surgery (e.g. POISE Trial Investigators, 2006; PROTECT Investigators, 2011) [52,53].

Since treatment allocation is concealed there will be no risk of conscious or unconscious selection bias on the part of the researchers, as can occur when it is possible for researchers to predict treatment assignment. Further, minimization will be used to ensure prognostic balance between intervention groups for relevant patient factors. The minimization approach evaluates the marginal distribution of each prognostic variable and then sums over the variables as each patient is assigned to a treatment group. Unlike random permuted block within strata, minimization works toward minimizing the total imbalance for all factors together instead of considering mutually exclusive subgroups [26] which may be considered an additional strength.

One limitation of the FAITH trial is the inability to blind patients, surgeons, and outcome adjudicators. Patients cannot be blinded as the two devices leave unique scarring patterns and surgeons cannot be blinded as the devices are easily discernible. It was planned that outcome adjudicators would be blinded to study treatment arm by digitally superimposing cancellous screws and the sliding hip screw on the same x-ray image for the assessments of quality of fracture reduction, fracture healing, infection, and avascular necrosis. However, after piloting this approach we found that it was still possible to often correctly identify the true treatment, and that difficulties with adjudication were created because of too much bone being covered. Therefore this approach was found to be infeasible for adjudicating and as a result outcome adjudicators will not be blinded to study treatment during their assessment of patients’ radiographic characteristics and quality of surgery. However, as revision surgery (the primary outcome) is objective, it is expected that a lack of blinding will not introduce significant threats to validity. To limit the minimal risk of bias that unblinded adjudication can create, a CAC will be used and consensus will be required on all adjudication events.

There is inconclusive evidence within the literature regarding operative method of internal fixation for femoral neck fractures. Prior evidence has suggested sliding hips screws may be superior to cancellous screws, but these findings have been based on studies with small sample sizes and low events numbers [25]. Advocates of multiple cancellous screws focus upon its superior torsional stability, limited disruption of femoral head blood supply, and minimally invasive insertion, while proponents of the sliding hip screw believe its superior biomechanical properties and greater fracture stability in osteoporotic bone should decrease the need for revision surgery. Despite the evidence suggesting the superiority of sliding hip screws, 90% of surgeons preferred cancellous screws over sliding hip screws for treating undisplaced fractures and 68% preferred cancellous screws over sliding hip screws in treating displaced fractures. Definitively identifying the optimal surgical approach to the initial management of femoral neck fractures has enormous potential to improve the lives of hundreds of thousands of individuals who suffer these injuries each year. The relative simplicity and ease of application of the proposed interventions will have wide applicability where trauma burden and disability from hip fractures is high. This trial will not only change current orthopaedic practice, but will set a benchmark for the conduct of future orthopaedic trials.

## Competing interests

We have no competing interests to disclose.

## Authors’ contributions

The Writing Committee [MB (Chair), MS, PJD, EHS, MJH, KJ, SL, LT, SW, SS, TS, GG] assumes responsibility for the overall content and integrity of the manuscript. *Study concept and design:* MB, MS, PJD, GG, MJH, KJ, SL, EHS, LT, SW. *Drafting of the manuscript:* MB, MS, SS, TS. *Critical revision of the manuscript for important intellectual content:* MB, MS, PJD, GG, MJH, KJ, SL, EHS, LT, SW. *Obtained Funding:* MB, MS, PJD, GG, MJH, KJ, SL, EHS, LT, SW. *Study Supervision:* MB, MS, PJD, GG, MJH, KJ, SL, EHS, LT, SW. All authors read and approved the final manuscript.

## Pre-publication history

The pre-publication history for this paper can be accessed here:

http://www.biomedcentral.com/1471-2474/15/219/prepub
